# Progress in Surgery of the Brain and Nerves

**Published:** 1904-01-30

**Authors:** 


*(To be continued.)*

### PROGRESS IN SURGERY OF THE BRAIN AND NERVES.

Two Cases of Cerebral Tumour Successfully Removed by Operation.—Pye-Smith[1] contributes this paper. The cases were diagnosed by the physicians, one tumour being situated in front of and the other behind the fissure of Rolando. In the first case a man of 50 had gradually been losing power in his left hand and forearm for over two years. He also experienced occasional cramp-like twitchings in the muscles of the forearm. About nine months before admission his sight began to be affected, and three months later the left lower limb became weak. About this time he began to experience slight giddiness and later had slight loss of control over his bladder. Headache commenced a month ago after a fall. There had been no fits, no loss of consciousness, no vomiting, and there was no history of syphilis. On admission there was drooping of the left eyelid, pupils equal, hemianopia, both optic discs swollen, almost complete loss of all movements of left forearm and hand, with wasting of interossei and short muscles of thumb, tactile sensation delayed, heat sensation absent, movements of the left foot slow and feeble, slight tremor of calf muscles, patellar tendon reflex increased, no ankle clonus, Babinski's sign present, sensation delayed and imperfect. Right upper and lower limbs normal. Is able to walk with a stick. He was treated for three weeks with large doses of iodide of potash and mercurial inunction, but got steadily worse, especially as regards the mental condition, which from being feeble on admission became childish. Operation being decided on, a square-shaped osteoplastic flap, having its centre corresponding to the centre of the fissure of Rolando, was raised and turned down. Trephine holes were made at the four angles, and the intervening bone divided with a Gigli's saw. The patient's pulse becoming weak at this stage the opening of the dura mater was deferred for two days and the flap replaced. At the second operation the dura was incised in a line corresponding with the flap and turned down. A portion of the brain about the centre of the exposed area was of darker colour than normal and abnormally hard on palpation. The posterior edge was well defined and in front of the Rolandic fissure. The growth was enucleated with the finger, commencing behind. In front it was adherent to the dura, which was removed with it. The tumour was about the size of a hen's egg. The dural flap was stretched back in position, a piece of gold leaf being placed over the deficiency and the osteoplastic flap replaced. The tumour was a spindle-celled sarcoma originating in the dura. Though somewhat collapsed after the operation, the patient recovered in a few days, and the sensation and motion in the affected parts slowly improved, power returning very slowly in the left forearm. The mental state also improved. About three months after the operation he had an attack of convulsive twitching in the left upper limb and face. These attacks were repeated, and about a month later, fearing a return of the growth, an exploratory operation was performed, the old wound being opened and more bone removed in a forward direction. There was no sign of growth, but the brain was adherent to the bone where the gold leaf had come away. It was freed, and a fresh piece of gold leaf placed over the exposed brain, and the wound closed. After this operation he lost the power he had previously regained in the left upper limb. He also had occasional slight convulsive attacks as before. He recovered, however, sufficiently to resume his work a month after leaving the hospital, improving in all respects except in the use of his left hand and arm. The second case was that of a man aged 37 who was admitted to hospital suffering from paralysis of the left arm and leg. On the previous night he had fallen down and lost consciousness, and on recovering the paralysis was noticed. The same day the affected parts twitched violently at intervals. Two years ago he fell down unconscious and remained so for 14 hours, but there was no paralysis or twitching. Apart from this he had always had good health. On admission all the organs were healthy; there was complete paralysis of the left arm; sensation normal and no wasting; the left leg was partially paralysed; no loss of sensation or wasting; increased knee-jerk and ankle clonus present; continuous fibrillary twitching of muscles of calf during examination; chronic convulsions affecting the left side beginning in neck; no loss of consciousness. He remained in hospital 12 days, the convulsive attacks becoming less frequent and finally ceasing, and power returned in the paralysed parts. He remained well for a month, when he again lost consciousness and fell in an epileptiform fit. He recovered from this, but had two more similar attacks at intervals of about six weeks, the latter followed by a series of convulsions. These attacks began with slow flexion of left hand upon the wrist, then of forearm upon the arm followed by action of similar character in leg. There were clonic spasms of whole of left side, and the head and eyes were deviated to the left. He was quite conscious and tried to speak. No headache, vomiting, or optic neuritis were present, and there was no history of syphilis. He was given large doses of iodide of potash, but the attacks continued. Severe pain in the head appeared at this time, with irritability and other changes in the mental state. A diagnosis of tumour of the brain having been made and operation decided on, a large osteoplastic flap was turned down over the Rolandic area in the same way as in the previous case. The patient's condition being good, the dura was opened and a small tumour at once recognised—it was well defined, circular, and flattish, about three-quarters of an inch in diameter, and much firmer than the surrounding brain. The Rolandic fissure was immediately in front of it. There being no capsule to the tumour, it was excised with about a quarter of an inch of brain around it, and extending about half an inch in depth. Microscopically the growth was an endothelioma involving the cortex and immediately underlying the pia mater. The operation was well borne and the wound healed by first intention, but after some weeks fits of Jacksonian epilepsy began again, and a further exploratory operation was performed. The old wound was opened, and the incised dura found unhealed at one spot, with adhesions between it and the brain. These were separated, and a piece of gold leaf inserted as in the previous case. After this he had attacks of spasmodic movements in the left limbs, and up to the time of the report had frequent cramp-like attacks in left upper limb, but in other respects remained well five months after operation. Pye-Smith points out that the position of the growth in the second case is of interest in connection with recent experiments, showing that the motor area is limited posteriorly by the fissure of Rolando.

[1] Quarterly Med. Jour., Aug. 1903.

